# Spatial variability of nitrate pollution and its sources in a hilly basin of the Yangtze River based on clustering

**DOI:** 10.1038/s41598-021-96248-0

**Published:** 2021-08-18

**Authors:** Yuhuan Cui, Jie Wang, Shuang Hao

**Affiliations:** 1 School of Science, Anhui Agricultural University, Hefei, China; 2 School of Resources and Environmental Engineering, Anhui University, Hefei, China; 3 Anhui Province Key Laboratory of Wetland Ecosystem Protection and Restoration, Anhui University, Hefei, China

**Keywords:** Biogeochemistry, Environmental sciences

## Abstract

Nitrate (NO_3_^−^) pollution is a serious global problem, and the quantitative analysis of its sources contributions is essential for devising effective water-related environmental-protection policies. The Shengjin Lake basin, located in the middle to lower reaches of the Yangtze River in China was selected as the research area in our study. We first grouped 29 surface water samples and 33 groundwater samples using cluster analysis, and then analyzed potential nitrate sources for each dataset of δ^15^N–NO_3_^−^ and δ^18^O–NO_3_^−^ isotope values by applying a Bayesian isotope-mixing model. Our results show that the nitrogen pollution in the surface-ground water in the study area seriously exceeded to class V of the Environmental Quality Standard of Surface Water of China. The NO_3_^−^ in surface water from the mid-upper reaches of the drainage basin mainly originates from soil nitrogen (SN) and chemical fertilizer (CF), with contribution rates of 48% and 32%, respectively, and the NO_3_^−^ in downstream areas mainly originates from CF and manure and sewage (MS), with contribution rates of 48% and 33%, respectively. For the groundwater samples, NO_3_^−^ mainly originates from MS, CF, and SN in the mid-upper reaches of the drainage basin and the northside of Dadukou near the Yangtze River, with contribution rates of 34%, 31%, and 29%, respectively, whereas NO_3_^−^ in the lower reaches and the middle part of Dadukou mainly originates from MS, with a contribution rate of 83%. The nitrogen conversion of surface water in lakes and in the mid-upper reaches is mainly affected by water mixing, while the groundwater and surface water in the lower plains are mainly affected by denitrification. The method proposed in this study can expand the ideas for tracking nitrate pollution in areas with complex terrain, and the relevant conclusions can provide a theoretical basis for surface and groundwater pollution control in the hilly basin of Yangtze River.

## Introduction

Nitrogen is an essential element for all living organisms and is a primary nutrient that restricts life on Earth^1^. Nitrate (NO_3_^−^) accounts for the highest proportion of all forms of nitrogen, however, the water with an extremely high NO_3_^−^ concentration poses a serious threat to human health as it can cause methemoglobinemia, digestive-system cancer, and other disorders or diseases^2–4^. Furthermore, research has been conducted on the influence of environmental changes and human activities on aquatic ecosystems by tracking nitrate sources and the nitrate-conversion process^5–8^. Therefore, distinguishing the sources of nitrates and clarifying their biogeochemical processes are essential for water-resources management at national and regional scales^9,10^.

Identification of nitrate sources in the surface water and groundwater has become a complex task owing to the involvement of different geochemical processes and potentially multiple nitrate sources^11–15^. Traditional method of determining nitrate sources relies on combining land-use types with the hydrogeochemical theory. However, this method can only analyze the impact of different land use types on nitrate concentration qualitatively, and lack quantitative analysis of the contribution of different land use types to nitrate. With the improvement in nitrate isotopic testing methods, analysis of stable isotopes in water is one of the effective methods for tracing pollution sources. Previous studies have proved that the δ^15^N and δ^18^O dual isotope-tracer technology such as the Bayesian isotope-mixing (MixSIAR) model provides an important method for tracking nitrate pollution in surface water and groundwater. It has become an effective tool for identifying nitrate sources in aquatic ecosystems and biogeochemical-cycle mechanisms^16–19^.

Affected by the combined influences of precipitation^20^, hydrogeological conditions^21,22^, land-use^23,24^, and hydrological processes^15,25^, δ^15^N–NO_3_^−^ and δ^18^O–NO_3_^−^ in water exhibit large spatiotemporal differences. Even minor changes in their values may cause a considerable change in the source contribution ratio. However, current researches prefer to track nitrate sources of the whole basin and few researches consider differences among nitrate sources within a basin. Therefore, our paper opted to analyze the spatial heterogeneity of nitrate concentrations and the proportions of different nitrate sources in watersheds, based on cluster analysis**.** The MixSIAR model was selected to improve the decomposition accuracy of the possible nitrogen sources in the study area, using the dual isotope values of δ^15^N–NO_3_^−^ and δ^18^O–NO_3_^−^.

Shengjin Lake basin, which encompasses middle and lower reaches of the Yangtze River (YZR), was selected as the study area here. It is mainly composed of Zhangxi River watershed (ZXH), Tangtian River watershed (TTH), Dadukou watershed (DDK) and Shengjin Lake (Lake). First, land-use types, water ions, and isotope values for NO_3_–N and oxygen were combined to analyze spatial characteristics of NO_3_^−^ in the surface water and groundwater of the basin. Then, all the water samples were grouped through cluster analysis and analyzed the contribution percentages and uncertainties of NO_3_^−^ sources for each group of water samples by applying the MixSIAR model. The purposes of our study are: (1) explore the spatial differences of nitrate ions as well as nitrogen- and oxygen-isotope values in the surface water and groundwater of the basin; (2) explore the effects of nitrification and denitrification processes in the basin’s different waterbodies on the nitrate-ions concentrations; (3) explore possible sources of nitrate pollution for each group of water samples. The information resulting from this study can provide effective ways for the accurate attribution of nitrate sources and effective control of nitrogen pollution, and also help to formulate appropriate management methods and effective water-quality protection policies for the Yangtze River basin.

## Results

### Spatial variations of hydro-chemical and nitrate isotopic parameters

We generated statistics for NO_3_^−^ and NH_4_^+^ concentrations in 29 surface water (DDKs, TTHs, ZXHs, and Lake) samples and 33 groundwater (DDKd, TTHd, and ZXHd) samples collected in April 2017 (Table 1; Fig. S1). NO_3_^−^ concentrations in surface water of different regions can be represented as ZXHs > TTHs > Lake > DDKs. Due to the low precipitation during the sampling period, the base flow of ZXHs and TTHs in the upper reaches of the basin is mainly recharged by groundwater, which greatly increases the impact of groundwater with a high concentration of NO_3_^−^ on surface water. Burns and Kendall obtained similar conclusions for a forest basin in the eastern United States^26^. Shengjin Lake was replenished mainly by the Zhangxi and Tangtian Rivers during the sampling period, so NO_3_^−^ concentration was considerably higher. The TN, NO_3_^−^ values in surface water exceed to class V of the Environmental Quality Standard of Surface Water of China (GB3838-2002), but not exceed the World Health Organization guidelines (the maximum value of nitrate in drinking water is 50 mg/L). The average values of NO_3_^−^ and NH_4_^+^ concentrations in the groundwater samples can be represented as DDKd > ZXHd > TTHd. The average values of TN and NO_3_^−^ in groundwater are higher than that in surface water, which all exceed to the Class V standard (GB3838-2002).

**Table 1:** Hydrochemical parameters of Shengjin Lake basin in April 2017.

	TN(mg/L)	NO_3_^-^(mg/L)	NH_4_^+^(mg/L)	δ^15^N–NO_3_^−^(‰)	δ^18^O–NO_3_^−^(‰)
Lake(n = 6)	1.86 ± 0.18	1.63 ± 0.21	0.21 ± 0.05	5.59 ± 1.08	3.28 ± 0.78
ZXHs(n = 11)	2.25 ± 0.58	2.08 ± 0.57	0.16 ± 0.06	4.73 ± 1.91	5.67 ± 1.43
ZXHd(n = 3)	5.56 ± 2.17	5.41 ± 2.13	0.14 ± 0.04	14.23 ± 4.20	8.11 ± 3.21
DDKs(n = 6)	1.91 ± 0.14	1.41 ± 0.36	0.63 ± 0.11	6.31 ± 2.30	1.42 ± 1.47
DDKd(n = 21)	7.50 ± 4.78	7.28 ± 4.81	0.17 ± 0.06	14.66 ± 5.26	8.27 ± 3.36
TTHs(n = 6)	2.16 ± 0.35	2.01 ± 0.40	0.25 ± 0.21	4.71 ± 2.07	5.77 ± 1.67
TTHd(n = 9)	5.30 ± 1.84	5.11 ± 1.88	0.13 ± 0.05	9.71 ± 6.05	7.51 ± 4.10

DDKs and DDKd represent surface water and groundwater of DDK, respectively. TTHs and TTHd represent surface water and groundwater of TTH, respectively; ZXHs and ZXHd represent surface water and groundwater of ZXH, respectively; Lake represents water in Shengjin Lake.

**Figure 1:**
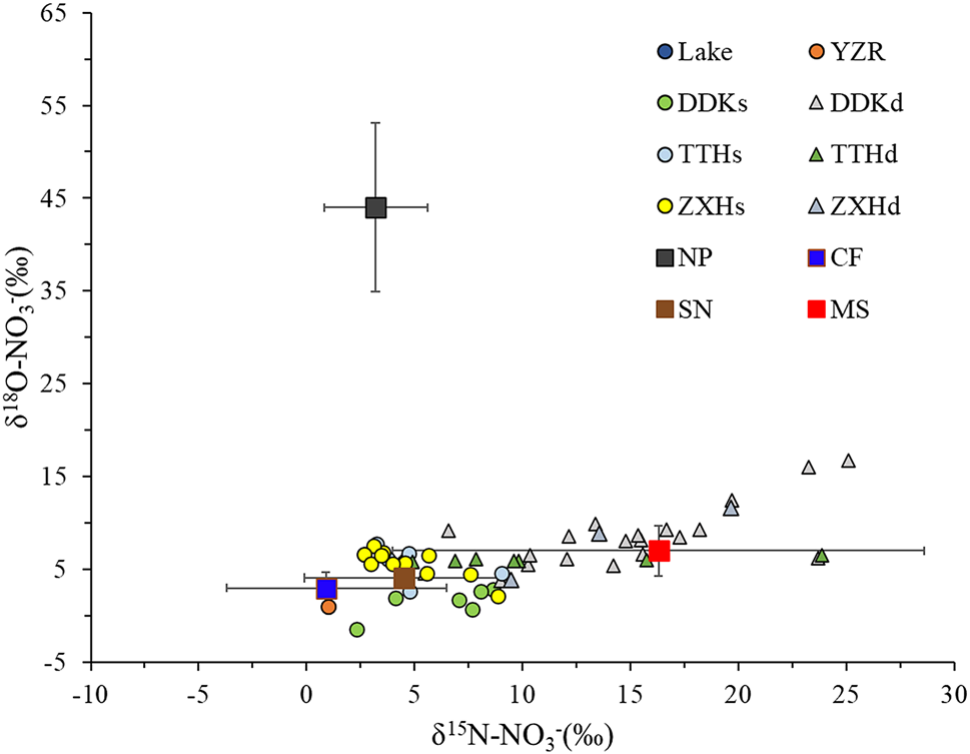
Cross plot of d^15^N and d^18^O values in the water samples of Lake, YZR, DDKs, DDKd, TTHs, TTHd, ZXHs and ZXHd. The ranges of isotopic composition of various sources including nitrogen fertilizer (CF), atmospheric precipitation (NP), soil organic nitrogen (SN), and manure/sewage (MS).

The range of δ^15^N–NO_3_^−^ in surface water and groundwater is + 2.3‰ ~  + 9.0‰, + 4.3‰ ~  + 25.1‰, and the range of δ^18^O–NO_3_^−^ is − 1.5‰ ~  + 7.7‰ and + 3.8‰ ~ respectively + 18.2‰, respectively (Fig. 1, Figure S2). The proportions of δ^15^N–NO_3_^−^ and δ^18^O–NO_3_^−^ in the surface water in Shengjin lake basin during the sampling period mainly reflect the values range for the soil, derived mostly from MS as the source^27^, which indicates that the NO_3_^−^ in the surface water is possibly sourced from MS in the soils. The proportion of δ^15^N–NO_3_^−^ and δ^18^O–NO_3_^−^ in the groundwater is mainly lay in the value range of MS sources, also supporting MS as the possible source of NO_3_^−^ in groundwater. Also, N derived from CF is not obvious in the δ^15^N–NO_3_^−^ and δ^18^O–NO_3_^−^ data, however, considering that CF can enter groundwater through the soil or the biological processes in the unsaturated zone (such as nitrification and possible denitrification) and then enter the river (where surface water and groundwater interacts), the contribution of CF as a source of N should not be ignored^28^.

### Water samples grouped by clustering

Cluster analysis is usually selected for the classification of hydrogeochemical data^29,30^. The nitrate concentrations, isotope values and the sources in Shengjin lake basin exhibit significant spatiotemporal variation, which caused by the combination of precipitation, hydrogeology, land-use, and hydrological processes. Considering the spatial heterogeneity of nitrate sources, the squared Euclidean distance as a proxy of similarity and the squared deviation method were used to group all the water samples according to δ^15^N–NO_3_^−^ and δ^18^O–NO_3_^−^ values, to improve the decomposition accuracy of nitrate sources.

29 surface water samples were divided into two groups (A and B) by hierarchical cluster analysis (Figure S3). Group A (Htt1-Htt, Hzx4-Hzx11) corresponds to surface water from the mid-upper reaches of the Zhangxi and Tangtian Rivers, and group B (Htt5, Htt6, Hzx1-Hzx3, S1-S6, H11, H12, H21, H31, H32 and H41) corresponds to lake water, surface water in the lower reaches of the Zhangxi and Tangtian Rivers, and surface water in Dadukou, which are most heavily polluted by agricultural nonpoint-source pollution and living point-source pollution.

33 groundwater samples into two groups (C and D) also divided through hierarchical cluster analysis (Figure S4). Group C (DD1, D1-D7, D11-D13, D31, D41, D45, and D48) corresponds to groundwater in the mid-upper parts of the Tangtian River watershed, as well as near the Yangtze River on the northern side of Dadukou. Group D (D8, D9, DD2, DD3, D14, D21-D23, D32-D36, D42-D44, D46, and D47) mainly corresponds to groundwater in the lower drainages of the Zhangxi and Tangtian Rivers, the middle of Dadukou, and near Shengjin Lake. The above clustering results of water samples were evaluated by the goodness of variance fit (GVF). The GVF values of group A and B were 0.73 and 0.72, respectively, and the values of group C and D were 0.88 and 0.86, respectively, all greater than 0.7, indicating that the clustering results are acceptable.

### Quantitative analysis of nitrate sources

After grouping the water samples by cluster analysis, the MixSIAR model was applied to quantified the contribution rates of possible nitrate sources to nitrate in water (Table 2), according to the range of isotope ratios of different nitrate sources (Table S1). The value ranges of isotope ratios for CF and SN in Table S1 are from the measurement in our study and the value ranges for NP and MS are referenced the study results of Zhang et al.^31,32^.

**Table 2:** Average contribution rates and deviations of nitrate sources in four water samples groups.

Groups		Contribution rates of nitrate sources (Mean ± SD)/%			
		CF	MS	NP	SN
Surface water	A	32.4 ± 13.9	9.6 ± 4.3	10.0 ± 2.2	48.0 ± 18.2
	B	48.0 ± 8.2	33.0 ± 6.1	2.2 ± 1.2	16.7 ± 10.6
Groundwater	C	31.3 ± 16.2	33.7 ± 5.7	5.5 ± 2.5	29.5 ± 21.1
	D	4.7 ± 3.4	83.3 ± 6.8	6.1 ± 3.6	5.9 ± 5.0

NO_3_^−^ in surface water from the mid-upper reaches of the Zhangxi and Tangtian Rivers (group A) is derived mainly from SN and CF. NO_3_^−^ in the surface water from Shengjin Lake, the lower reaches of the Zhangxi and Tangtian Rivers, and the Dadukou area (group B) is derived mainly from CF and MS (Table 2). Therefore, SN and CF are the main sources of NO_3_^−^ in the mid-upper reaches of the basin, while the main sources of NO_3_^−^ in the lower reaches and lake areas are CF and MS. During fieldwork, we found that the lower reaches of the basin are densely populated with villages and towns as well as farms, but also that domestic (residential) sewage and manure from free-range livestock are directly discharged into the river, which presumably leads to higher NO_3_^−^ concentration in the river, it is also the reason why the MS contribution rate in group B is significantly higher than that of group A.

In the mid-upper watersheds of the Zhangxi and Tangtian Rivers as well as on the northside of Dadukou near the Yangtze River (group C), the NO_3_^−^ in groundwater samples mainly comes from MS, CF, SN, and NP. In the lower reaches of the Tangtian and Zhangxi Rivers and in the middle of Dadukou (group D), the NO_3_^−^ in the groundwater mainly derives from MS, with a contribution rate of 83%.

## Discussion

### Influence of biogeochemical processes on nitrate pollution

As the major forces driving the nitrogen cycle, denitrification and nitrification have an important regulatory effect on nitrogen loss and N_2_O emissions. The δ^18^O value produced by nitrification has been estimated − 10.3‰ to − 4.5‰ based on the δ^18^O–H_2_O^33^ and as + 23.5‰ based on the δ^18^O–O_2_ value^34^. Among the atoms needed for nitrification, one third come from surrounding O_2_ and the rest are from surrounding H_2_O^35^.

**Figure 2:**
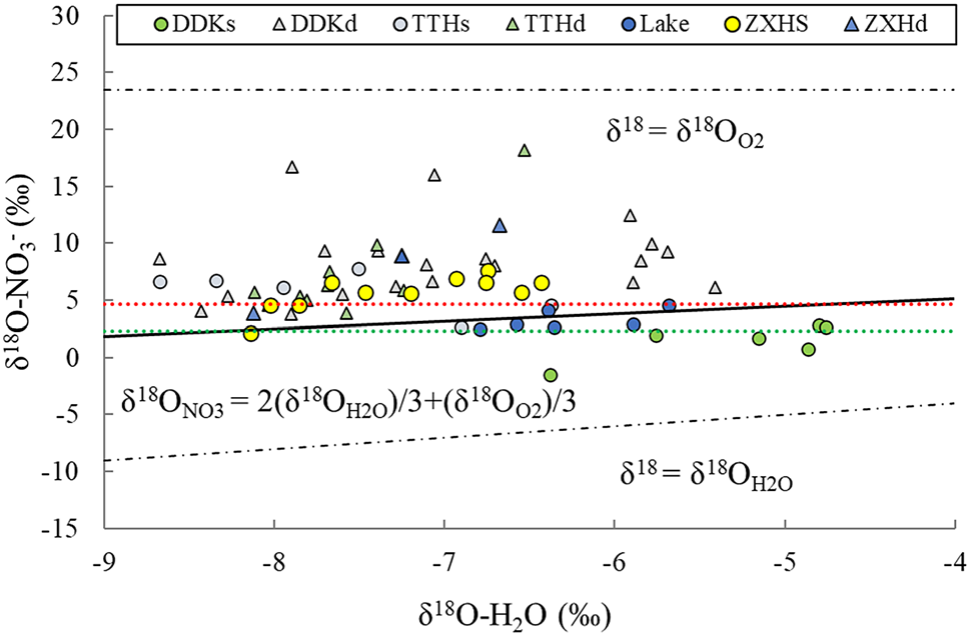
Variations in δ^18^O–H_2_O and δ^18^O–NO_3_^−^ values in Shengjin Lake basin. Note: The three lines represent the theory lines in different conditions. The red and green dashed lines indicate the maximum and minimum theoretical values of δ^18^O–NO_3_^−^, respectively.

The δ^18^O values for the water samples in the Shengjin Lake basin are within the range from − 8.7‰ to − 4.8‰, while the δ^18^O–NO_3_^−^ values produced by nitrification are theoretically within + 2.03‰ to + 4.63‰. The δ^18^O–NO_3_^−^ values for all the samples approximate theoretical values (Fig. 2). The δ^18^O–NO_3_^−^ values for most water samples from the Tangtian and Zhangxi Rivers are higher than the upper limit of the theoretical range of the values, because the isotopic composition of nitrate produced by nitrification is more affected by soil nitrification than river water^7,36,37^. The δ^18^O–NO_3_^−^ values of some surface water samples in the DDK are lower than the theoretical lower limit of nitrification, which may be caused by the addition and exchange of NO_3_^−^ in the unevaporated soil water to produce more oxygen^37^.

**Figure 3:**
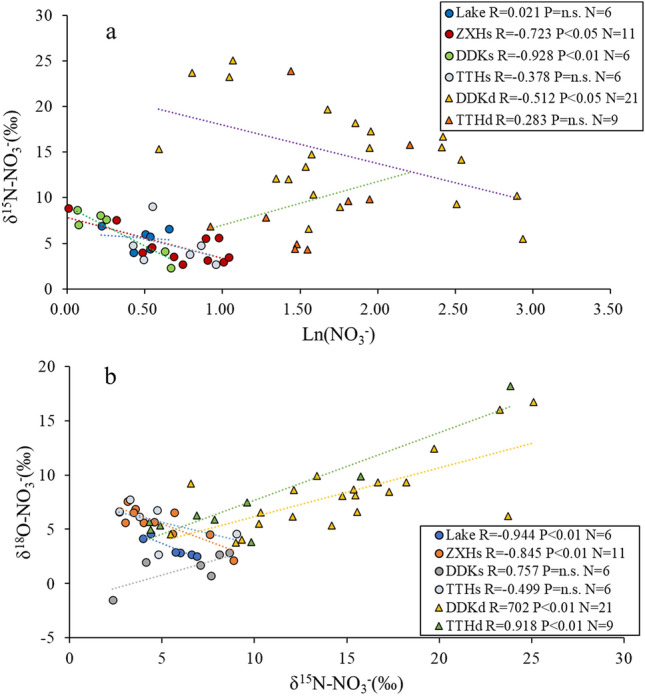
Relationships of δ^15^N–NO_3_^−^ versus ln (NO_3_^−^), δ^18^O–NO_3_^−^ versus δ^15^N–NO_3_^−^ values in Shengjin lake basin (R is Pearson correlation; P indicates that the correlation is significant at the 0.01 level (2 tails); N represents the number of statistical samples; n.s. = nonsignificant and *p* > 0.05).

The relationships between the δ^15^N–NO_3_^−^ and ln (NO_3_^−^) concentrations, the δ^15^N–NO_3_^−^ and δ^18^O–NO_3_^−^ values in the water samples are further analyzed (Fig. 3a,b), to explore the influence of denitrification on NO_3_^−^ concentrations. We found that the δ^15^N–NO_3_^−^ and ln (NO_3_^−^) concentrations in the DDKs, DDKd, and ZXHs showed a significant negative correlation (*P* < 0.01), which suggests that denitrification is the main nitrogen conversion process^38^. In the Lake and ZXHs water samples, the δ^15^N–NO_3_^−^ and δ^18^O–NO_3_^−^ values also showed a significant negative correlation (*P* < 0.01), indicating that no heavy-isotope enrichment and denitrification occurred^7,36,39^. In the DDKs, DDKd and TTHd samples, the δ^15^N–NO_3_^−^ and δ^18^O–NO_3_^−^ values showed a significant positive correlation (*P* < 0.05), indicating possible denitrification. Thus, obvious denitrification occurred in the DDKs and DDKd water sample. However, we found no obvious sign of denitrification in the TTHs water sample. The reason can be described as the Tangtian River watershed is in the mountainous area of the upper reaches of Shengjin Lake basin where the exchange and mixing of surface water and groundwater have weakened the isotope signal for denitrification. Similarly, denitrification in the Lake water samples is not obvious, which could be explained by partial recharge by various tributaries and by groundwater. The mixing effect of different waterbodies is strong and the sign of denitrification was weak, which similarly concluded by Xia et al.^40^. The denitrification signals in the DDKs, TTHd, and ZXHs water samples are not consistent, which may also be an effect of nitrification and the mixing of surface water and groundwater.

### Relationship between conductivity and nitrate pollution

The complex nitrate sources in surface–groundwater can be determined by water chemical composition. The conductivity (COND) is mainly determined by the species, concentration and temperature of the ion in the water, and it is related to the exchange rate of the water, the lithology of the rock formation, and human pollution input. Other Studies implied that the high positive correlation between NO_3_^−^ and COND appeared in water with high eutrophication^41^. Moreover, COND has been identified as the main indicator for detecting domestic fecal pollution, industrial sewage and other emissions^42^. Therefore, COND is used as an indicator of NO_3_^−^ pollution source in our study.

**Figure 4:**
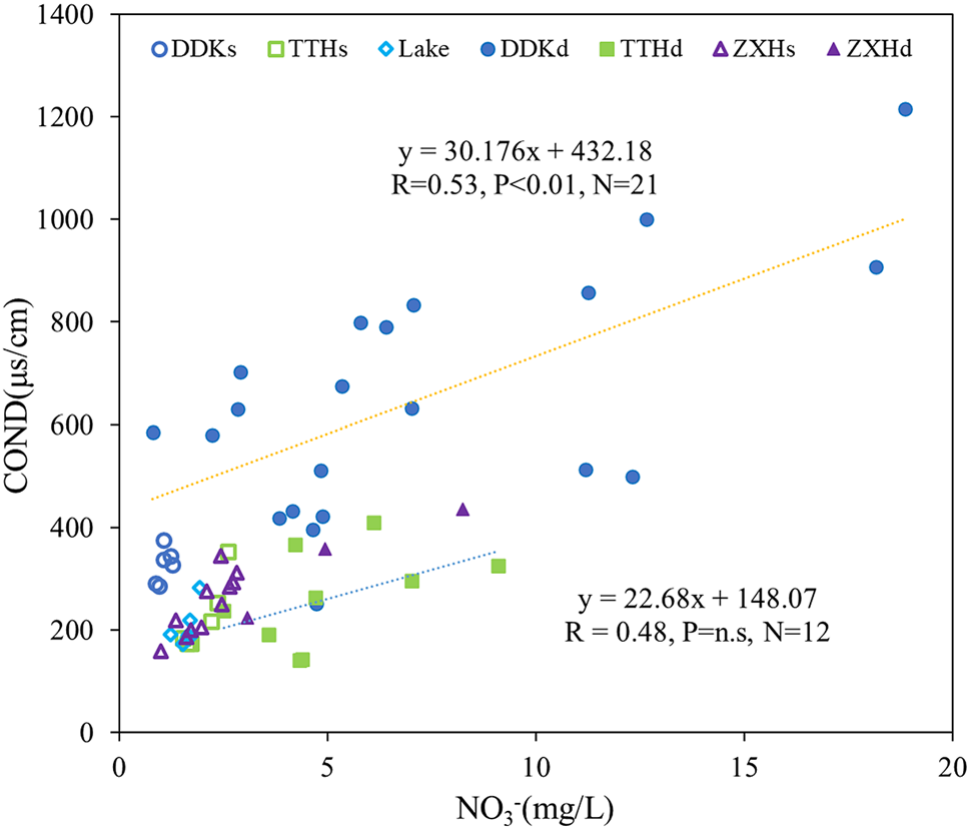
The relationships between COND vs. NO_3_^−^ in surface-ground water (R is Pearson correlation; P indicates that the correlation is significant at the 0.01 level (2 tails); N represents the number of statistical samples; n.s. = nonsignificant and p > 0.05).

Figure 4 shows that the COND in DDKd is positively correlated with NO_3_^−^ concentration (R = 0.57, *P* < 0.01, N = 21), and the COND and NO_3_^−^ concentration are much larger than other regions, indicating that manure/sewage is the main nitrogen pollution source in DDK. The COND in TTHd and ZXHd are not significantly correlated with NO_3_^−^ in the upper reaches of the watershed (R = 0.48, P = n.s., N = 12), but the values of COND and NO_3_^−^ are significantly lower than DDKd, indicating that the multiple sources of nitrogen pollution exist. DDKs have a larger COND value, but a lower NO_3_^−^ concentration, indicating that the denitrification process may have occurred in the river, resulting in the decrease in NO_3_^−^. TTHs, ZXHs and Lakes in the mid-upper reaches of the watershed have the lower COND values and higher NO_3_^−^ concentration, indicating that surface water may be recharged by groundwater with higher NO_3_^−^ concentration, which caused the NO_3_^−^ concentration of surface water increased. Therefore, the relationship between COND and NO_3_^−^ can explain the contribution of manure/sewage to nitrogen sources in surface and groundwater.

### Influence of land use on nitrate sources

Many studies have shown that there are significant differences in land use patterns in different regions, causing the differences in nitrate pollution and their sources^43,44^. The primary land use in DDK, the lower reaches of TTH and ZXH is agricultural land. As the main fertilizers used in agriculture are nitrogen fertilizers, such as compound fertilizers, urea and ammonium nitrate-based, excessive fertilization and low utilization rates are common^45^, which has led to CF becoming the main source of nitrate in the surface water of Group B. Moreover, although the agricultural area accounts for about 15%, chemical fertilizer is still one of the main nitrate sources in the mid-upper reaches of the basin, which is related to the agricultural planting model. Affected by the complex terrain, agriculture in the mid-upper reaches of the basin is dominated by scattered planting, causing problems such as excessive application of nitrogen fertilizer and low utilization rate. This is also one of the serious problems faced by rural areas in China^46^.

In addition, MS is the largest contributor to NO_3_^−^ in groundwater in the lower reaches of the basin, the contribution ratio of which is much larger than those in the mid-upper reaches. Due to the lack of sewage pipeline system, most of the sewage in rural toilets of China is directly discharged into rivers and lakes, posing a serious threat to the local ecosystem and environment. In addition, sewage discharged from large-scale livestock farms in suburbs and villages is another major source of pollution^47^. Therefore, MS is also the main source of NO_3_^−^ pollution in groundwater in Group D.

The forest coverage rate in the mid-upper reaches of the basin gets to 69%, and the coverage rate of farmland and residents only accounts for about 23%. The nitrate in the forest-dominated watershed mainly comes from soil nitrification^16^, which caused the SN in the middle and upper reaches of the watershed contributes a very high rate of surface water and groundwater.

However, although the mid-upper watershed areas have less agricultural and residential land and thus lower nitrate concentration in theory, the average values of nitrate concentration in surface water and groundwater reached 2.0 and 5.0 mg/L (Table 1).

## Conclusions

Based on the analysis of the spatial distribution of nitrate pollution in the Shengjin lake basin, our paper first uses cluster analysis to group water samples, and then uses the MixSIAR model to explore the spatial differences of nitrate pollution sources within the basin. The NO_3_^−^ of surface water in the mid-upper reaches of the drainage basin mainly originates from SN and CF, with the contribution rates of 48% and 32%, respectively, whereas that of surface water in the downstream reaches mainly originates from CF and MS, with contribution rates of 48% and 33%, respectively. The NO_3_^−^ in the groundwater samples from the mid-upper parts of the drainage basin and on the northern side of Dadukou close to the Yangtze River mainly originates from MS, CF, and SN, with the contribution rates of 34%, 31% and 29%, respectively; whereas that from the lower parts of the basin and from the middle part of Dadukou mainly originates from MS, with a contribution rate of up to 83%.

Nitrogen conversion of surface water in lakes and in the mid-upper part of the basin is governed mainly by water mixing, and that of groundwater and surface water in the lower plains is influenced mainly by denitrification.

The clustering method was firstly applied to group water samples, and then the MixSIAR model was used to analyze the contribution rate of nitrates from different sources in the water quantitatively, which proved to be an effective method for tracing nitrogen sources in the watersheds. In the mid-upper reaches of the hilly basin along the Yangtze River, nitrate pollution control is mainly achieved through the strategies such as limiting the use of pesticides and fertilizers; while in the plains at the lower reaches of the basin, with the density population, it is mainly through the promotions of ecological agriculture development to control the use of agricultural fertilizers, the construction of rural sewage discharge pipelines and sewage treatment facilities, to reduce the impact of chemical fertilizers and fecal sewage on nitrate pollution.

In recent years, nitrate pollution in rural watersheds has attracted the widespread attention in China. The relevant pollution control policies such as ecological agriculture, centralized discharge of rural sewage, and renovation of rural toilets have been formulated. Since Shengjin lake basin in our research has the ideal representation in terms of topography, land use, hydrology, social economy, etc., the conclusions and nitrate pollution control strategies drawn above can also be extensively implemented in the middle and lower reaches of the Yangtze River.

## Materials and methods

### Study area

**Figure 5:**
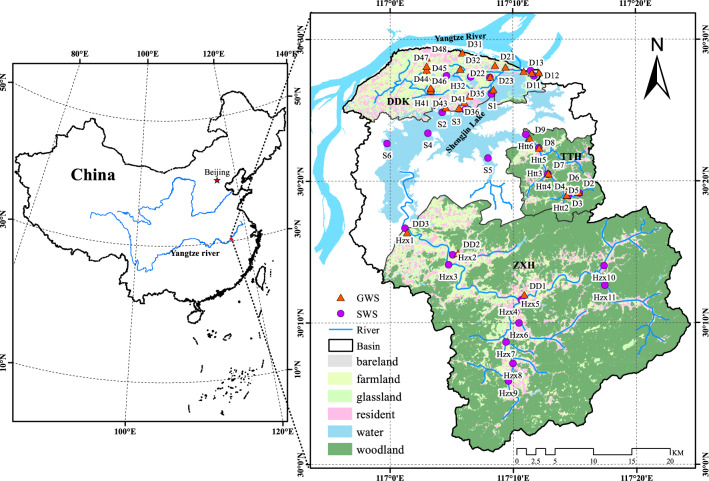
Distribution of three watersheds and water-sampling sites in Shengjin Lake basin.

Shengjin Lake basin, with an area of 1445.2 km^2^, is on the southern side of the middle and lower reaches of the Yangtze River. Surface runoff from the east, south, and west directions merges to flow into Shengjin Lake (Fig. 5). The Zhangxi River and Tangtian River are the two main rivers in the upper reaches of the basin that drain into the Shengjin Lake. They flow down from sparsely populated, dense woodland hilly areas (Zhangxi River watershed (ZXH) and Tangtian River watershed (TTH)) over a significantly undulating terrain. The area of these two watersheds is 827 km^2^ and 107 km^2^, and the population is about 59, 600 and 25,000, respectively^45^. Farmland area accounts for 15% and 13% of these two watersheds, and residential and woodland areas both account for 9% and 69%, respectively. In the lower reaches of these two watersheds, poultry, fish and shrimp breeding are the main economic industries, while the middle and upper reaches are dominated by agricultural planting.

The Dadukou watershed (DDK), in the lower reaches of the drainage basin, with the area of about 157 km^2^ and the population of about 80,300, located at the junction of Shengjin Lake and the Yangtze River (Fig. 1). As the main populated area and an intensive agricultural-planting area in the basin^45^, the DDK is greatly affected by human activities. The proportions of farming and residential areas are 37%, 22%, respectively, while the woodland area accounts for only approximately 1%. The main economic industries in this watershed are metal manufacturing, clothing processing, waterfowl and aquaculture and other breeding industries, and the agricultural planting mode is intensive agriculture.

### Field-sample collection

The hydrological survey of Shengjin Lake basin was performed April 3rd to 16th, 2017, and 62 sampling sites were set up, including 29 surface water samples (SWS) and 33 groundwater samples (GWS). For DDK, we obtained six surface water samples (DDKs), including three subsamples (H31, H32, and H41) from stagnant rivers and three subsamples (H11, H12, and H21) from flowing rivers; 21 distributed groundwater (DDKd) samples (D11–D48) were also obtained. For TTH, we obtained six surface waters (TTHs) samples (Htt1–Htt6) from the Tangtian River and nine groundwater (TTHd) samples (D1–D9). For ZXH, we obtained 11 surface waters (ZXHs) samples (Hzx1–Hzx11) from the Zhangxi River and three groundwater (ZXHd) samples (DD1–DD3). We also obtained six surface water (Lake) samples (S1–S6) from Shengjin Lake. Except for the three water samples from stagnant rivers, surface water samples from rivers and lakes were collected from places that having high-velocity water movement at a > 5 m distance from the shore. For the groundwater samples, except for D31 (field-irrigation wells), the rest samples were all from wells in residential areas, and the samples were collected at the bottom of the wells using a miniature pump to reduce the effect of water-depth differences on isotope values. We used a real-time kinematic positioning system (RTK Stonex S3) to determine the three-dimensional coordinates of each sampling site, the water-surface altitude was also calculated, which can be used to determine the water level and flow direction. Also, rainwater samples from April 2015 to July 2016 were collected monthly to measure the values for δ^2^H–H_2_O, δ^18^O–H_2_O, δ^15^N–NO_3_^−^, and δ^18^O–NO_3_^−^. A 2-L separatory funnel was used to collect rainwater, and paraffin oil was added to prevent evaporation.

After rinsing polyethylene bottles with water, 500-ml water samples were collected at each sampling site. After passing the water samples through a 0.45-μm glass-fiber filter, they were subjected to measurement in the laboratory to obtain amounts of chemical components include total nitrogen, inorganic nitrogen, and cations and anions. Furthermore, 20-ml water samples were enclosed in treated-headspace vials for δ^15^N–NO_3_^−^ and δ^18^O–NO_3_^−^ isotopic analysis, and 2-ml water samples were stored in GC brown-glass bottles in a 4 °C refrigerator for later δ^2^H–H_2_O and δ^18^O–H_2_O testing.

Unfertilized soil (sampled at 10-cm depth) and chemical fertilizers (CF) from five sites representing different land-use types were also collected. The soil samples were prepared according to Rock et al.’s method^48^, and the fertilizer samples were prepared using Heaton et al.’s method^49^. We used a 0.45-μm glass-fiber membrane to filter each sample, divided the sample into a 20-ml headspace bottle, and refrigerated it for later δ^15^N–NO_3_^−^ and δ^18^O–NO_3_^−^ testing.

### Water-chemistry and isotope values measurements

Temperature, dissolved oxygen, conductivity (COND), and pH of the water samples were measured on site with a handheld multiparameter meter (YSI professional plus, West Lyme, made in USA). Total nitrogen concentration was determined through alkaline potassium persulfate digestion ultraviolet spectrophotometry, and the NO_3_–N was measured by Dionex ICS-1500 ions, with a difference between anion and cation charge balances of < 5%. The content of ammonia nitrogen (NH_4_^+^–N) and nitrite nitrogen (NO_2_^−^–N) were determined through spectrophotometry.

Laboratory tests of H–O isotopes and NO_3_–N–O isotopes were processed in the Environmental Stable Isotope Laboratory, Chinese Academy of Agricultural Sciences. H–O isotopes were pyrolyzed through thermal-conversion elemental analysis and processed into H_2_ and CO, following which the isotope values were measured. The Vienna Standard Mean Ocean Water was set as the standard sample, the accuracy of the δD and δ^18^O measured values reached 0.2% and 0.01%, respectively.

Isotope values of NO_3_–N and O are determined by converting NO_3_–N to N_2_O using specific denitrifying bacteria^27^. Using USGS32, USGS34, and USGS35 as standard samples, we calibrated the measured gas using the two-point calibration method, and N– and O– isotope values of N_2_O were obtained by TraceGas combined with isotope mass spectrometry.

### MixSIAR model

The MixSIAR model, based on the Dirichlet distribution, builds a logical prior distribution under the Bayesian framework. It can be used to estimate the contribution percentages of different nitrogen sources^50^ and is expressed as

1 $$ \begin{gathered} X_{ij} = \mathop \sum \limits_{k = 1}^{k} P_{k} \left( {S_{jk} + C_{jk} } \right) + \varepsilon_{jk} \hfill \\ S_{jk} \sim N\left( {\mu_{jk} ,\omega_{jk}^{2} } \right) \hfill \\ C_{jk} \sim N\left( {\lambda_{jk} ,\tau_{jk}^{2} } \right) \hfill \\ \varepsilon_{jk} \sim N\left( {0,\sigma_{j}^{2} } \right) \hfill \\ \end{gathered} $$

where X_ij_ is δ value of isotope *j* in mixture *i*; *i* = 1, 2, 3… *N*, and *j* = 1, 2, 3… *J*; *P*_*k*_ is proportion of source *k* as estimated by the model; *S*_*jk*_ is δ value of source *k* isotope *j*, obeying the normal distribution of mean value *μ*_*jk*_ and variance *ω*_*jk*_; *Cjk* is fractionation coefficient of source *k* isotope *j*, obeying normal distribution with mean value *λ*_*jk*_ and variance *τ*_*jk*;_ *ε*_*jk*_ is residual error, representing variance that cannot be quantified among other mixtures with mean value zero and standard deviation *σ*_*j*_.

The MixSIAR model was created and run by the R software package MixSIAR v.3.1.10^51^, to quantify the contributions of nitrate from four different sources (fertilizer, rainfall, sewage, and soil). The N-isotope characteristics of sewage are similar to those of manure, so these two sources were considered as one source (MS) in our study^52^.

### Statistical analysis

Cluster analysis, based on the similarity and closeness of datasets, is a statistical method for grouping data, is usually selected for the classification of hydrogeochemical datasets^29,30^. The Ward’s method of hierarchical clustering with Squared Euclidean Distance was applied to explore the grouping of the water samples, and the Goodness of Variance Fit (GVF)was used to assess its accuracy.

2 $$ {\text{GVF}} = 1 - {\text{SDCM}}/{\text{SDAM}} $$

where SDAM represents the sum of squared deviations from the array mean; SDCM represents the sum of squared deviations about class mean.

All data analyses and plottings were performed using the SPSSv.22 statistical software package, Excel 2010, and the Windows operating system.

### Ethical approval and consent to participate

Not applicable.

### Consent for publication

Not applicable.

## Supplementary Information

Supplementary Information.

## Data Availability

Data and material access are not available.
