# Supplementary material for: Spatial variability of nitrate pollution and its sources in a hilly basin of the Yangtze River based on clustering
Source: Sci Rep. 2021 Aug 18;11:16752. doi: 10.1038/s41598-021-96248-0 (PMC8373962; doi:10.1038/s41598-021-96248-0)
Supplement: Supplementary file 1 — Supplementary Information. [file 41598_2021_96248_MOESM1_ESM.pdf]

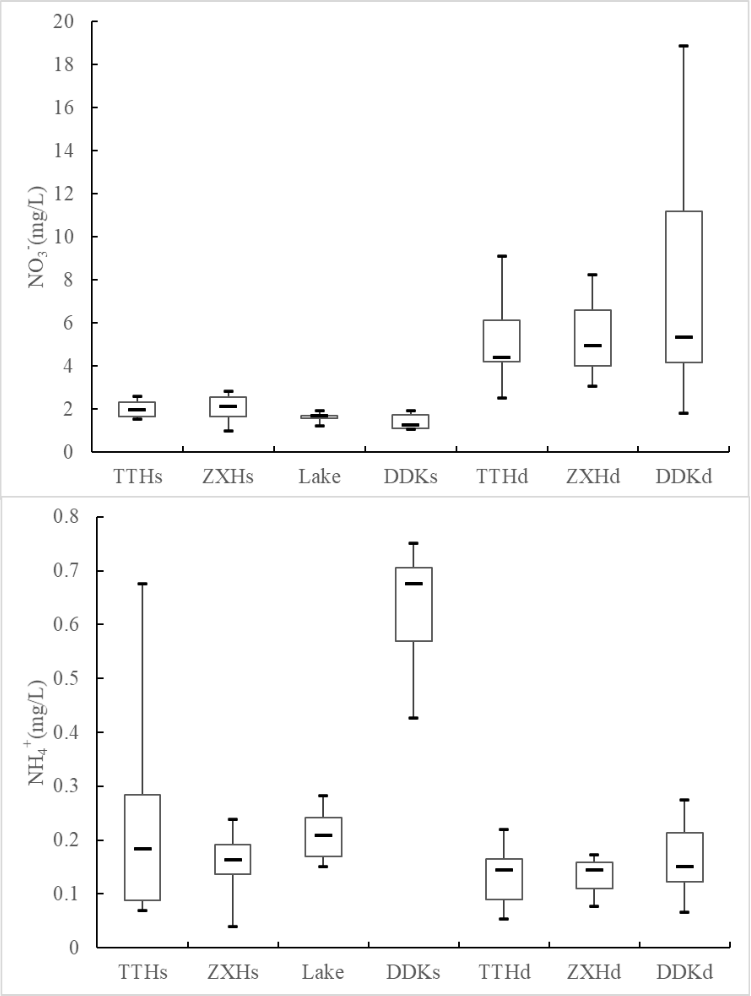

Figure S1 Box diagrams of NO_3^-, NH_4^+ concentrations in different sampling areas

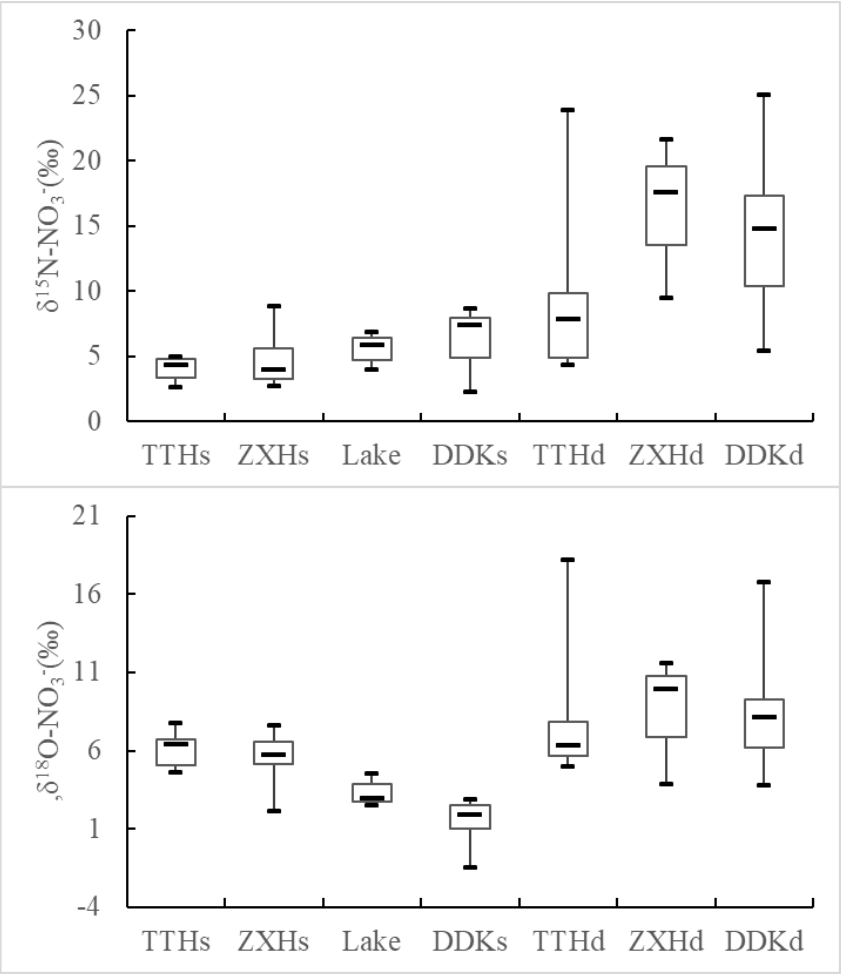

Figure S2 Box diagrams of $\delta^{15}N–NO_3^-$, $\delta^{18}O–NO_3^-$ concentrations in different sampling areas

Note: DDKs and DDKd represent surface water, groundwater of DDK, respectively. TTHs and TTHd represent surface water and groundwater of TTH, respectively; ZXHs and ZXHd represent surface water and groundwater of ZXH, respectively; Lake represents surface water in Shengjin Lake; Yangzi represent Yangtze River water

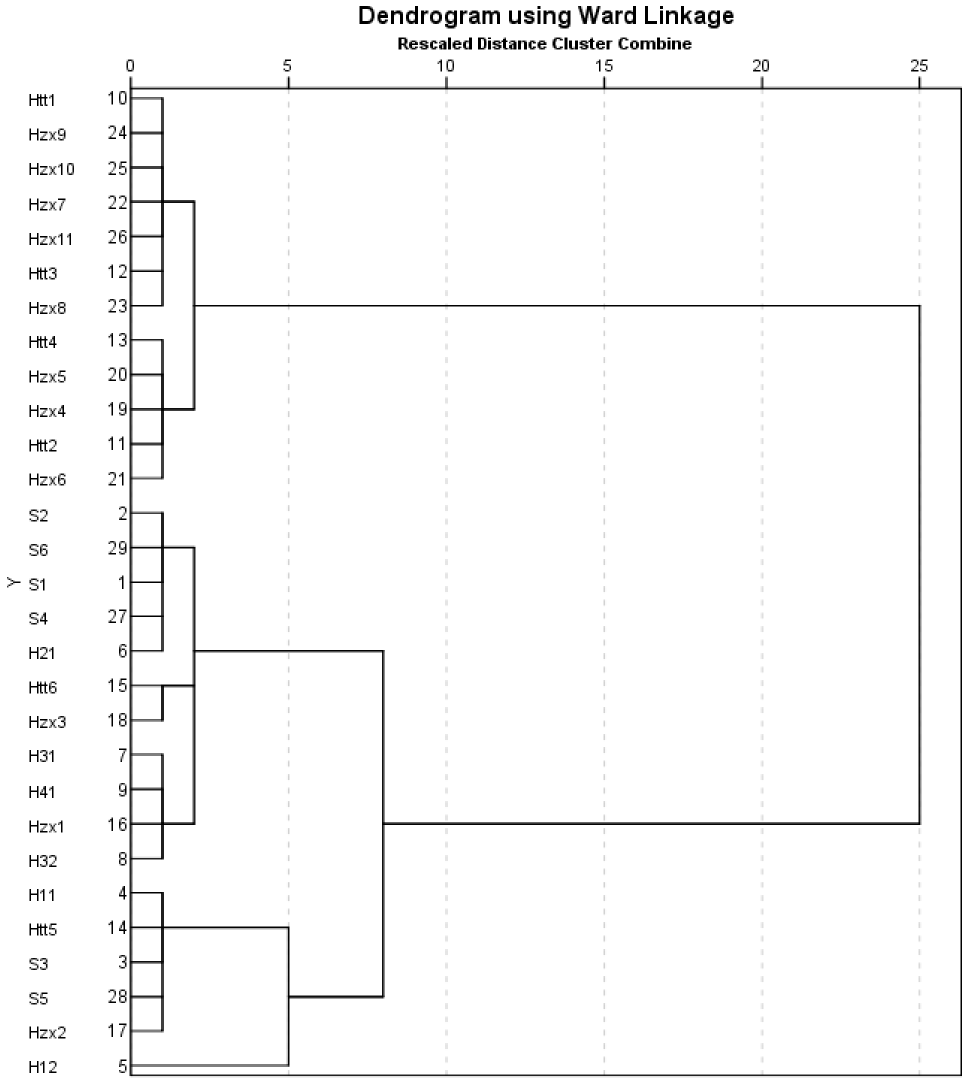

Figure S3. Dendrogram of surface water obtained by hierarchical cluster analysis

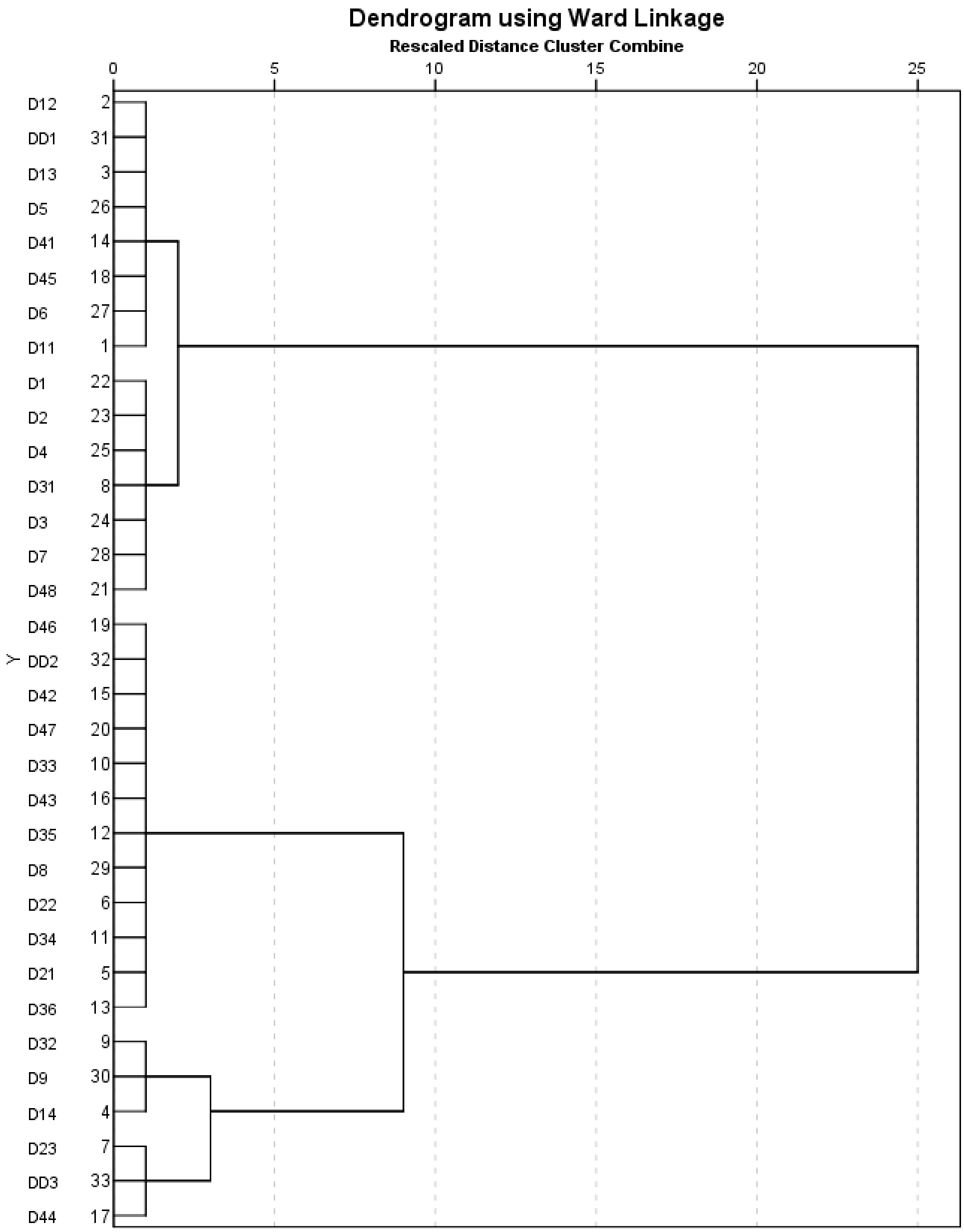

Figure S4. Dendrogram of groundwater obtained by hierarchical cluster analysis

Table S1 Value ranges of $\delta^{15}N$ and $\delta^{18}O$ corresponding to different NO_3^- sources (NP, CF, SN and MS)

Source	$\delta^{15}N$ (‰)			$\delta^{18}O$ (‰)		
	Max	Min	Mean±SD	Max	Min	Mean±SD
NP	+6.6[a]	-3.9[a]	+3.20 ± 2.40[b]	+61.4[a]	+26.3[a]	+44.00 ± 9.10[b]
CF	+4.7[a]	−6.1[a]	+0.90 ± 2.50[b]	+5.6[a]	−2.9[b]	−2.00 ±0.46[b]
SN	+7.2[a]	+1.4[a]	+4.00 ± 1.5[b]	+5.6[a]	−0.9[a]	+4.08 ± 0.33[b]
MS	+23.1[a]	+7.4[a]	+16.30 ± 5.70[a]	+12.3[a]	+2.8[a]	+7.00 ± 2.70[a]

Note: NP is the precipitation nitrogen source, SN is the soil nitrogen source, CF is the chemical fertilizer nitrogen source, and MS is the manure/sewage nitrogen source. [a] is the value of reference 1, 2, and [b] is the measured data.

References

1. Zhang Y, Shi P, Li F D, et al. Quantification of nitrate sources and fates in rivers in an irrigated agricultural area using environmental isotopes and a Bayesian isotope mixing model. Chemosphere, 2018, 208: 493-501
2. Zhang M, Zhi YY, Shi J C, et al. Apportionment and uncertainty analysis of nitrate sources based on the dual isotope approach and a Bayesian isotope mixing model at the watershed scale. Science of the Total Environment, 2018, 639:1175-1187
